# Early Hippocampal i-LTP and LOX-1 Overexpression Induced by Anoxia: A Potential Role in Neurodegeneration in NPC Mouse Model

**DOI:** 10.3390/ijms18071442

**Published:** 2017-07-05

**Authors:** Adriana Lo Castro, Michela Murdocca, Sabina Pucci, Anna Zaratti, Chiara Greggi, Federica Sangiuolo, Virginia Tancredi, Claudio Frank, Giovanna D’Arcangelo

**Affiliations:** 1 Department of Medical System, University of Rome Tor Vergata, Rome 00133, Italy; a.locastro@libero.it (A.L.C.); tancredi@uniroma2.it (V.T.); 2 Department of Biomedicine and Prevention, University of Rome Tor Vergata, Rome 00133, Italy; miky.murdi@hotmail.it (M.M.); sabinapuc@yahoo.it (S.P.); chiaragreggi@gmail.com (C.G.); sangiuolo@med.uniroma2.it (F.S.); 3 CNMR, Istituto Superiore di Sanità Roma, Rome 00161, Italy; anna.zaratti1983@libero.it (A.Z.); claudio.frank@iss.it (C.F.)

**Keywords:** NPCD, neurodegeneration, i-LTP, LOX-1

## Abstract

Niemann-Pick type C disease (NPCD) is an autosomal recessive storage disorder, characterized by abnormal sequestration of unesterified cholesterol within the late endo-lysosomal compartment of cells. In the central nervous system, hypoxic insults could result in low-density lipoprotein (LDL) oxidation and Lectin-like oxidized LDL receptor-1 (LOX-1) induction, leading to a pathological hippocampal response, namely, ischemic long-term potentiation (i-LTP). These events may correlate with the progressive neural loss observed in NPCD. To test these hypotheses, hippocampal slices from Wild Type (WT) and *NPC1*^−/−^ mice were prepared, and field potential in the CA1 region was analyzed during transient oxygen/glucose deprivation (OGD). Moreover, LOX-1 expression was evaluated by RT-qPCR, immunocytochemical, and Western blot analyses before and after an anoxic episode. Our results demonstrate the development of a precocious i-LTP in *NPC1*^−/−^ mice during OGD application. We also observed a higher expression of LOX-1 transcript and protein in *NPC1*^−/−^ mice with respect to WT mice; after anoxic damage to LOX-1 expression, a further increase in both *NPC1*^−/−^ and WT mice was observed, although the protein expression seems to be delayed, suggesting a different kinetic of induction. These data clearly suggest an elevated susceptibility to neurodegeneration in *NPC1*^−/−^ mice due to oxidative stress. The observed up-regulation of LOX-1 in the hippocampus of *NPC1*^−/−^ mice may also open a new scenario in which new biomarkers can be identified.

## 1. Introduction

Niemann-Pick type C disease (NPCD) is a rare autosomal recessive neurodegenerative disease. Mutations in *NPC1* (*MIM 607623*) or *NPC2* (*MIM 601015*) genes cause a wide spectrum of neuropsychiatric deficits such as movement disorders, vertical supranuclear palsy of the gaze, epilepsy, psychiatric syndromes, and cognitive damage, ranging in severity from specific learning disorders up to mental retardation or dementia. The above symptoms are related to several pathological modifications, which reflect the endolysosomal lipid accumulation and affect the central nervous system (CNS), with the involvement of both gray and white matter [1,2]. At the neuronal level, affected patients show a significant lipid accumulation. Similar to other neurodegenerative disorders, NPCD is classified among tauopathies for the presence of intracellular aggregates of tau proteins biochemically identical to those found in Alzheimer’s disease. These findings are synonymous with oxidative damage related to neurodegeneration [3]. The *NPC-1* gene codifies an integral membrane protein containing a sterol-sensing domain involved in low-density lipoprotein (LDL)-cholesterol (LDL-C) trafficking from endosome/lysosome to the plasma membrane. For this reason, NPC-1 probably plays a protective role in cholesterol intracellular accumulation, governing the balance between macrophage cholesterol import and export [4]. Cholesterol metabolism determines the presence of LDL in the brain. LDL consists of cholesterol and hundreds of phospholipid, molecules that may, under certain conditions, be promptly oxidized (oxLDL). oxLDL in atherosclerosis plays a role together with LOX-1 in mediating neuronal loss [5,6]. Moreover, neurons and brain regions, which are more prone to oxidative stress, are rich in LOX-1 receptors. Specifically a correlation between LOX-1 and neuronal apoptosis was reported [7]. Recently a strong link was reported between hypoxia and the induction of LOX-1 in HN33 neuronal cell lines [8]. It is well known that glutamate-mediated excitotoxicity, oxidative stress, inflammation, and apoptosis may be involved in the neuronal death processes after ischemia, leading to neurodegeneration [9,10]. The hippocampus is a cerebral area particularly susceptible to hypoxic insult that leads to neuronal damage. A brief ischemic insult (5 min) generates a pathological form of synaptic plasticity, known as ischemic long-term potentiation (i-LTP) [11], a synaptic mechanism that, by an increase of intracellular calcium, triggers apoptosis and is a well-known electrophysiological correlate of molecular apoptotic cell death [12]. Since an early and progressive neurodegeneration has been reported in NPCD, the aim of the present study was to characterize the increased susceptibility of hippocampal NPCD neurons to the ischemic insult, which is a transient oxygen/glucose deprivation (OGD) in vitro. This model has been extensively studied to clarify the pathophysiological bases of the epileptic activity and of neuronal post-stroke damage [12,13].

For this purpose, hippocampal slices from WT (wild type) and *NPC1*^−/−^ mice were prepared and the field potential recording was analyzed. Moreover LOX-1 expression at the transcript and protein levels was evaluated in both mice at basal conditions and after anoxia was experimentally induced.

## 2. Results

### 2.1. Precocious i-LTP in NPC1^−/−^ Hippocampal Slices

The population spike (PS) amplitude in slices of the hippocampal CA1 regions of *NPC1*^−/−^ and WT mice has been studied during OGD, when a 5 min single anoxic episode was applied, and then for the period of reperfusion with normal artificial cerebro-spinal fluid (ACSF) for 60 min. Every 10 s, the evoked spikes were recorded for about 20 min to ensure a stabilization of the signal.

**Figure 1:**
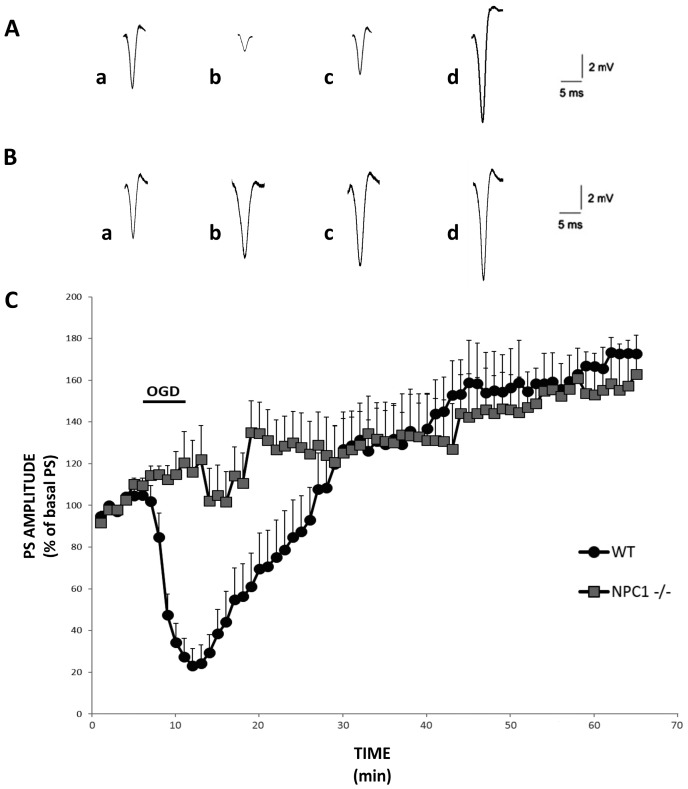
Effects of oxygen/glucose deprivation (OGD) in slices from wild type (WT) and *NPC1*^−/−^ mice on the CA1 hippocampal subfield. Representative population spike (PS) recorded during anoxia from (**A**) WT hippocampal slices and (**B**) *NPC1*^−/−^ slices; traces recorded (a) before anoxia, (b) at the end of OGD, and (c) 10 min and (d) 40 min after the OGD episode. Each trace represents the average of six recordings. (**C**) The percentage of the PS amplitude as a function of time during and after the OGD episode in WT (black circles, *n* = 13) and *NPC1*^−/−^ (gray squares, *n* = 11) mice. Each point in the plot is the mean ± SEM of the values from diverse slices. The PS amplitude, measured with a one-minute interval, represents the average of six recordings per minute. It is noted that at 5 min of OGD, the PS is significantly higher in the *NPC1*^−/−^ slices with respect to the WT (120 ± 10 versus 20 ± 10; *p* < 0.05). Early ischemic long-term potentiation (i-LTP) is induced 10 min after the anoxia episode in *NPC1*^−/−^ mice with respect to the WT slices (135 ± 10 vs. 65 ± 15; *p* < 0.05).

In control conditions (WT mice), the anoxic episode induced a significant decrease of the PS amplitude, followed by a complete recovery within 20 min. Reperfusion with ACSF for 25 min potentiated the synaptic transmission by about 30% to 40% compared to the basal values, and this increase was maintained for the rest of the time of recording (Figure 1A–C, black circles; *n* = 13). In *NPC1*^−/−^ slices, the reduction in PS amplitude failed to appear during the anoxic episode. Moreover, after 10 min of ACSF reperfusion, the PS amplitude was significantly higher compared to WT, indicating the insurgence of a precocious ischemic long-term potentiation (Figure 1B,C, gray squares; *n* = 11).

### 2.2. LOX-1 Analysis

**Figure 2:**
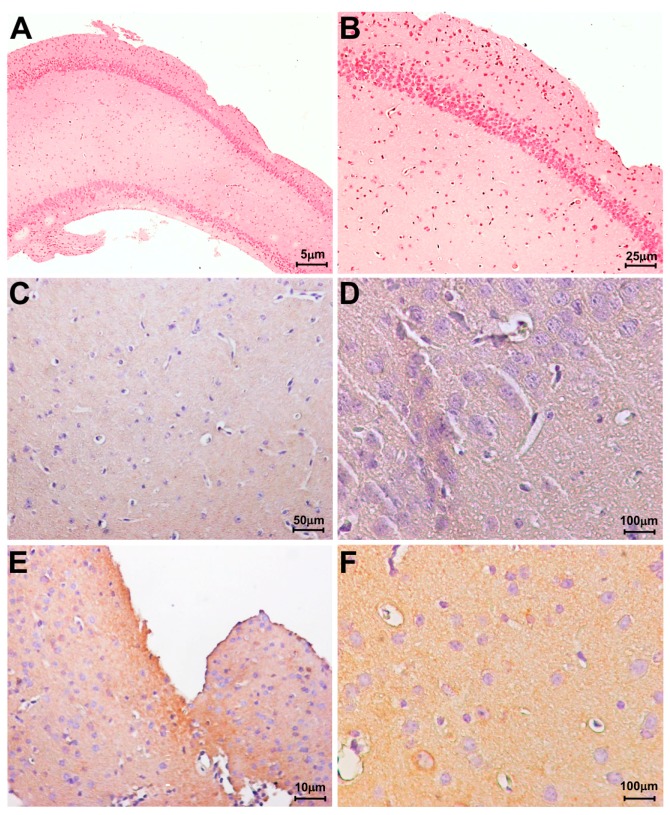
Immunohistochemical analysis of Lectin-like oxidized LDL receptor-1 (LOX-1) expression in hippocampal tissues. (**A**,**B**) haematoxylin eosin counterstain in hippocampal tissues from wild type mice; (**C**–**F**) immunohistochemistry for LOX-1; (**C**,**D**) A faint positive reaction for LOX-1 was observed in hippocampal tissues from wild type mice; (**E**,**F**) LOX-1 expression in *NPC1*^−/−^ hippocampal tissues.

In order to verify the possible differential expression of LOX-1, immunohistochemistry was performed on hippocampal tissues from WT and *NPC1*^−/−^ mice. As shown in Figure 2, in the hippocampal sections of *NPC1*^−/−^ mice, a higher and diffuse expression of LOX-1 was evident (Figure 2E,F) as compared to WT mice (Figure 2C,D).

**Figure 3:**
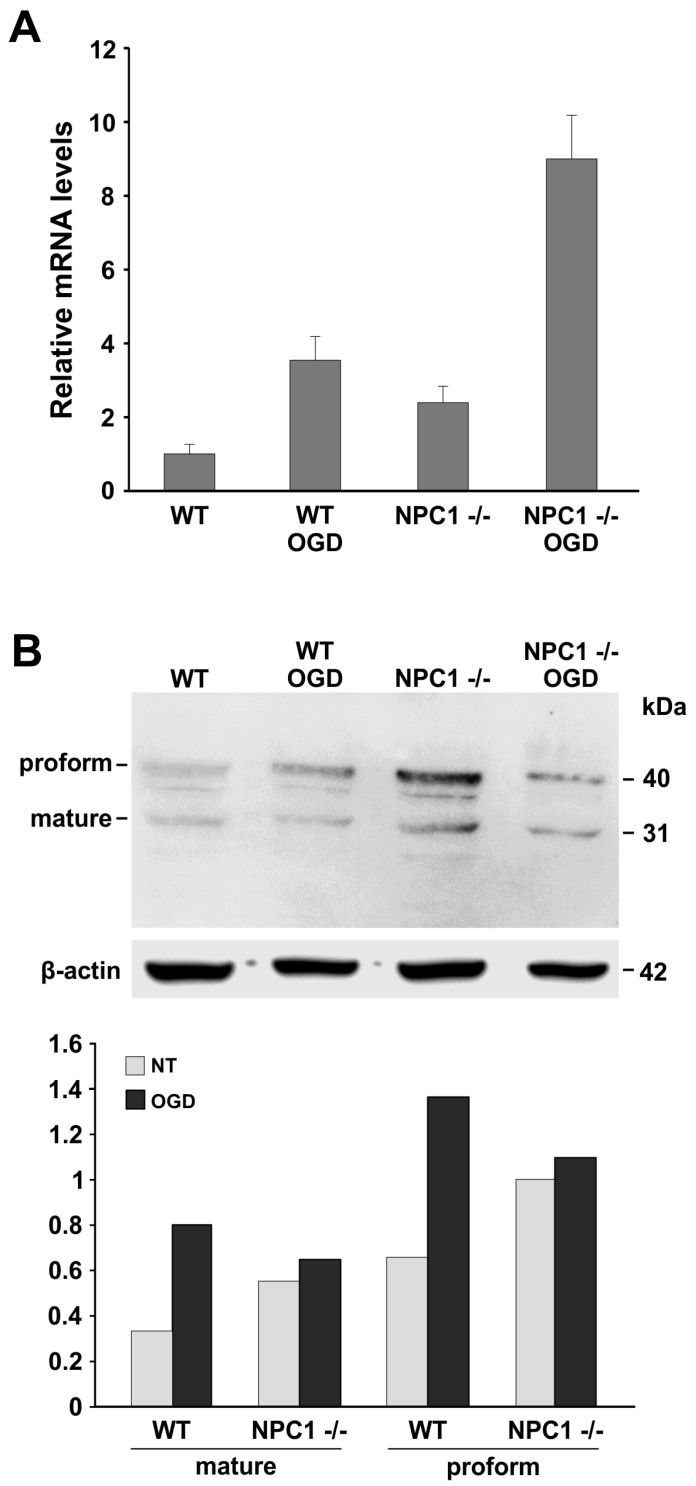
LOX-1 expression in WT and *NPC1*^−/−^ mice. (**A**) RT-qPCR analysis of LOX-1 expression in the hippocampi of WT and *NPC1*^−/−^ mice. (WT versus *NPC1*^−/−^ and WT OGD versus WT: *p* < 0.05; *NPC1*^−/−^ OGD versus *NPC1*^−/−^: *p* < 0.01). Values represent mean ± SEM. Data are representative of three independent replicates; (**B**) Western blot analysis of LOX-1 protein expression performed on the total protein extracts from hippocampal tissues before and after the anoxic insult in WT and *NPC1*^−/−^ mice. Proforms and mature forms of LOX-1 are indicated. β-actin levels are reported as housekeeping. Densitometric analyses of Western blot are reported in the bar graph as the meaning of three independent experiments. (Mature and proform values: WT versus *NPC1*^−/−^, WT OGD versus WT: *p* < 0.01; mature and proform: *NPC1*^−/−^ OGD versus *NPC1*^−/−^: *p* < 0.05). The analyses were carried out on mice tissue slices (*NPC1*^−/−^ *n* = 10 mice; WT *n* = 6 mice; *NPC1*^−/−^ OGD *n* = 10 mice; WT OGD *n* = 6 mice).

LOX-1 expression was evaluated by real time-qPCR in hippocampal slices obtained from WT and NPC mice before and after an induced anoxic episode (Figure 3A). A statistically significant increase of LOX-1 expression was detected in *NPC1*^−/−^ mice compared to WT mice. The deprivation of oxygen and glucose in *NPC1*^−/−^ mice hippocampus in vitro caused a marked increase in the levels of LOX-1 with respect to the untreated mice.

The LOX-1 protein level was investigated by Western blot analysis before and soon after damage (10 min) in order to verify the kinetic of LOX-1 protein induction. As shown in Figure 2B,C, LOX-1 was strongly overexpressed in *NPC1*^−/−^ mice as compared to WT mice. After the deprivation of oxygen and glucose, we observed a prompt increase of LOX-1 proform and mature form in WT mice; conversely, in *NPC1*^−/−^ mice, we observed a slight increase of protein levels.

**Figure 4:**
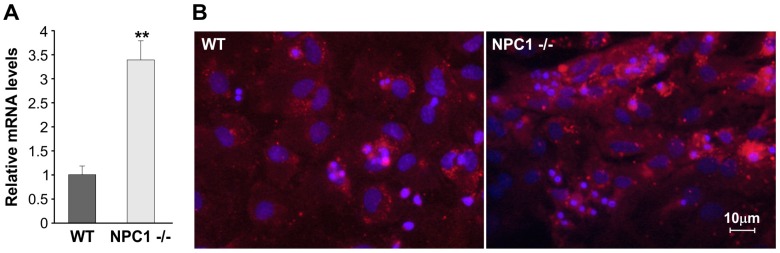
LOX-1 expression in hippocampal cells obtained from WT and *NPC1*^−/−^ mice. (**A**) RT-qPCR analysis. Values represent mean ± SEM. Data are representative of three independent replicates. (** *p* < 0.05); (**B**) Representative images of immunocytochemical analyses performed using anti LOX-1 antibody in cells obtained from WT and *NPC1*^−/−^ mice. Analyses were carried out on hippocampal cells obtained from the mice (*NPC1*^−/−^ *n* = 10 mice; WT *n* = 6 mice).

Furthermore, RT-qPCR and immunocytochemical analyses were performed on hippocampal cells obtained from WT and *NPC1*^−/−^ mice. The results again confirmed the difference of LOX-1 expression between WT and *NPC1*^−/−^ mice, as shown in Figure 4A,B.

## 3. Discussion

The most common pathological feature of NPCD is the progressive loss of neurons and the dysfunction of cells in the brain [14]. Since in NPCD an early and progressive neurodegeneration has been observed, the electrophysiological response to conditions similar to that induced by ischemic damage has been evaluated in the present paper. We found that, in slices of *NPC1*^−/−^ mice under anoxia-aglycemia, a characteristic response to OGD is observed: (1) a lack of disappearance of synaptic transmission during the OGD application; (2) an early ischemic LTP that developed after almost 10 min of reperfusion; (3) an increase in the expression of LOX-1 receptors both in basal conditions and after ischemia induction.

These results suggest an increased susceptibility to neurodegeneration in *NPC1*^−/−^ mice.

Several studies indicate that OGD in the hippocampal area induced neuronal death and that glutamate exerts a toxic action. Moreover it has been suggested that adenosine plays a pivotal role in neuroprotection when CNS is subjected to metabolic or traumatic stress [15,16].

When adenosine accumulates in the extracellular space during hypoxia, the depression of excitatory transmission is induced through the activation of presynaptic A1 receptors at glutamatergic terminals [17].

The persistence of synaptic transmission during OGD application in *NPC1*^−/−^ slices presumably could be explained by the observation that the concentration of adenosine in the extracellular space of the NPC1 mouse brain was decreased, inducing an enhancement of glutamatergic synaptic transmission in CA1 pyramidal neurons [18]. Hence NPC1 mice are more vulnerable because of a reduced adenosine neuroprotective effect.

The observation that i-LTP induced in slices of *NPC1*^−/−^ mice hippocampi appears earlier than in WT mice, validates the hypothesis that NPC1 mice are prone to neurodegeneration. There is experimental evidence that this aberrant form of synaptic activity depends on the activation of glutamate receptors and recognizes the same mechanisms and molecular pathways underlying the insurgence of physiological LTP, the basic mechanism of memory and learning processes [19].

The early i-LTP could be induced by an increased expression of NMDA and a transformation of the synapses presenting α-amino-3-hydroxy-5-methyl-4-isoxazolepropionic acid (AMPA) receptors shifting from a silent to an active state. In a previous paper [20], we reported that, in hippocampal slices from *NPC1*^−/−^ mice, glutamatergic neurotransmission is enhanced and that AMPA-induced calcium influx is increased in *NPC1*^−/−^ hippocampal neurons. We have suggested that, in *NPC1*^−/−^ mice, the cell surface exposure of the AMPA receptor could be increased due to cholesterol–sphingolipid deregulation that leads to an impairment of the AMPA receptor machinery of exo/endocytosis.

Several authors have described a correlation between membrane lipid composition and glutamate receptor functioning [21,22].

The alteration of lipid rafts, likely due to the well-known trafficking defect of cholesterol and glycosphingolipids (GSLs) and the accumulation of GSLs in NPCD, may be an important element in the induction of oxidative stress conditions [23].

Cholesterol metabolism finds the presence of LDL in the brain that could be oxidized, resulting in ox-LDL. The function of ox-LDL is well known, and many data establish the role played by ox-LDL in mediating neuronal loss. In addition, a recent letter by Liu [24] also consolidates the involvement of LOX-1 in neural injury, setting up a hub between LOX-1 and neurodegeneration.

To support this hypothesis, a quantification of LOX-1 expression was performed, comparing *NPC1*^−/−^ to WT mice before and after an ischemic insult. A significant increase of LOX-1 mRNA was detected in *NPC1*^−/−^ mice compared to WT mice, and this overexpression was greatly exacerbated after an anoxic induction. LOX-1 is mainly expressed in the endothelial cells of large arteries, smooth muscle cells, and monocytes/macrophages. It is an essential component of atherogenesis, and it induces endothelial dysfunction and the accumulation of foam cells [25]. Several studies have established a critical role for LOX-1 in cardiovascular diseases, but, together with other risk factors, it could influence the progression of neurodegenerative diseases during an individual’s life. However, the mechanisms through which this happens are poorly understood.

Data obtained by Western blot analysis point out a different kinetic of response to the damage between *NPC1*^−/−^ mice and wild type mice. The prompt induction of LOX-1 protein in WT mice indicates that, in the *NPC1*^−/−^ murine model, LOX-1 protein expression induction is delayed. The delayed induction of LOX-1 protein could be connected to hippocampal neuron damage in *NPC1*^−/−^ mice that seems to preserve the capability of LOX-1 mRNA to be induced in response to damage but suggests a postponement of LOX-1 mRNA traduction.

These data evidence a significant link between systemic risk factors and neurodegeneration. Our hypothesis is that increased levels of cholesterol due to the lack of NPC-1 protein and to oxidative stress consequent to NPCD disease up regulate the levels of ox-LDL receptor on cell membrane in brain tissues. Our results will allow further studies in order to evaluate LOX-1 levels as a biomarker for NPCD disease and to verify new therapeutic strategies.

## 4. Materials and Methods

### 4.1. Animals

We employed animals from a colony of *NPC1*^−/−^ mice that we established in our animal house and that is an experimental model of NPCD as the majority of the clinical aspects of the disease are displayed. Breeding pairs of BALB/cNctr-Npc1m1N/J (Stock Number: 003092) mice were delivered by Jackson Laboratories (Bar Harbor, MA, USA). In this strain, there is a spontaneous mutation in the NPC1 locus [26]. The breeding and keeping of the animals was performed following Italian Animal Care Committee rules (Ministerial Decree: Number 12/2005-Released on 4 February 2005). The genotypes of offspring animals born from heterozygous (male × female) mice were characterized following the Jackson Laboratories indications reported in the genotyping protocols database by polymerase chain reaction (PCR) [26]. Briefly, PCR was performed using the following primers; wild type forward: 5′-CTGTAGCTCATCTGCCATCG-3′, wild type reverse: 5′-TCTCACAGCCACAAGCTTCC-3′, mutant forward: 5′-GGTGCTGGACAGCCAAGTA-3′, and mutant reverse: 5′-TGAGCCCAAGCATAACTTCC-3′. Age-matched wild type mice served as the controls.

### 4.2. Extracellular Recordings in Mouse Hippocampus

Eleven genotypically characterized *NPC1*^−/−^ mice, as well as 13 wild type mice (six to eight weeks of age), were used, as stated by the EU Directive 2010/63/EU for animal experimentation. The hippocampal slices were prepared as previously described [20]. Cerebral ischemia has been reproduced, inducing anoxia/aglycemia by perfusing slices with glucose-free ACSF oxygen deprived (95% N_2_–5% CO_2_). In this oxygen-glucose deprived medium, glucose has been replaced with 10 mM sucrose to balance osmolarity. An ischemic period (5 min) was followed by reperfusion with normal ACSF (60 min).

PS recordings were made in the pyramidal layer of the CA1 region as previously described [20]. Data acquisition and storage were performed by a personal computer using a standard acquisition software (Axon, Foster City, CA, USA).

### 4.3. LOX-1 Expression Analysis

TRIzol Reagent (Invitrogen; Life Technologies Corporation, Carlsbad, CA, USA) was used to extract total RNAs from the hippocampal slices and cells following the manufacturer’s instructions, and then the RNAs were treated with DNase I (RNase-free Ambion, Life Technologies Corporation, Foster City, CA, USA). One μg of RNA was reverse transcribed with the High-Capacity cDNA Archive kit (Life Technologies Corporation, Foster City, CA, USA) and used in real-time reverse transcription (RT)–polymerase chain reaction (PCR). SYBR Green chemistry (Life Technologies Corporation) and specific primers for murine lox-1 and glyceraldehyde-3-phosphate dehydrogenase (*gapdh*) genes were used as follows; *lox*-1 forward: 5′-TCATCCTCTGCCTGGTGTTG-3′ and reverse: 5′-GTCAGATACCTGGCGTAATTG-3′, *gapdh* forward: 5′-ATGACATCAAGAAGGTGGTG-3′ and reverse: 5′-CATACCAGGAAATGAGCTTG-3′. Quantitative measurements were determined using the ΔΔ*C*_t_ method, and *gapdh* was used as the internal control.

### 4.4. Immunocytochemistry and Western Blot Analyses of LOX-1 in Hippocampus

Immunocytochemical analyses were performed on hippocampal cells derived from both WT and *NPC1*^−/−^ mice. Cells were isolated as previously reported [20] and fixed in 4% paraformaldehyde. Anti-LOX-1 primary antibody (1:100, Inc H19 Santa Cruz Biotecnology Inc., Santa Cruz, CA, USA) was used, and the cells were subsequently incubated for 1 h at room temperature with a specific secondary antibody and 4,6-diamidino-2-phenylindole (DAPI) (1:1000, Sigma-Aldrich, St. Louis, MO, USA).

Immunohistochemistry was assessed on hippocampal tissues fixed in 4% paraformaldehyde for 24 h and paraffin embedded. Three-micrometer thick sections were stained with hematoxylin and eosin (H&E). Serial 5 μm thick sections from formalin-fixed and paraffin-embedded specimens were immunostained for LOX-1 (Upstate Biotecnology, Lake Placid, NY, USA) following the streptoavidin-biotin method. Tissue staining was semi-quantitatively graded for intensity from negative (0) to strong (+++).

Western blot analysis was performed on the total protein extracts from the hippocampal tissues. The total proteins extract (10 μg) were equally loaded (Ponceau S-staining) on 10% SDS. Proteins were transferred to a PVDF membrane (Hybond P, Amersham GE Healthcare, Chalfont St. Giles, UK). Anti-LOX-1 (UpState Biotecnology) mouse monoclonal was used as the primary antibody. The filters were reprobed with anti-β-actin mouse monoclonal antibodies (Sigma-Aldrich) to normalize the protein levels. The filters were developed using an enhanced chemiluminescence system (ECL, Amersham-GE Healthcare).

### 4.5. Statistics

Data are expressed as mean measurements ± SEM, and *n* represents the number of slices studied. Data were statistically compared using Student’s *t*-test or the ANOVA test and were considered significantly different if *p* < 0.05 and *p* < 0.01. Excel 5.0 software was used for statistics and the generation of graphs.

## References

[1] Totenhagen J.W., Lope-Piedrafita S., Borbon I.A., Yoshimaru E.S., Erickson R.P., Trouard T.P. (2012). In vivo assessment of neurodegeneration in Niemann-Pick type C mice by quantitative T2 mapping and diffusion tensor imaging. J. Magn. Reson. Imaging.

[2] Zaaraoui W., Crespy L., Rico A., Faivre A., Soulier E., Confort-Gouny S., Cozzone P.J., Pelletier J., Ranjeva J.P., Kaphan E. (2011). In vivo quantification of brain injury in adult Niemann-Pick Disease Type C. Mol. Genet. Metab..

[3] Auer I.A., Schmidt M.L., Lee V.M.Y., Curry B., Suzuki K., Shin R.W., Pentchev P.G., Carstea E.D., Trojanowski J.Q. (1995). Paired helical filament tau (PHFtau) in Niemann-Pick type C disease is similar to PHFtau in Alzheimer’s disease. Acta Neuropathol..

[4] Millard E.E., Gale S.E., Dudley N., Zhang J., Schaffer J.E., Ory D.S. (2005). The sterol-sensing domain of the Niemann-Pick C1 (NPC1) protein regulates trafficking of low density lipoprotein cholesterol. J. Biol. Chem..

[5] Keller J.N., Hanni K.B., Markesbery W.R. (1999). Oxidized low-density lipoprotein induces neuronal death: Implications for calcium, reactive oxygen species, and caspases. J. Neurochem..

[6] Sugawa M., Ikeda S., Kushima Y., Takashima Y., Cynshi O. (1997). Oxidized low density lipoprotein caused CNS neuron cell death. Brain Res..

[7] Sawamura T., Kume N., Aoyama T., Moriwaki H., Hoshikawa H., Aiba Y., Tanaka T., Miwa S., Katsura Y., Kita T. (1997). An endothelial receptor for oxidized low density lipoprotein. Nature.

[8] Mao X., Xie L., Greenberg D.A. (2014). LOX-1 expression and oxidized LDL uptake and toxicity in the HN33 neuronal cell line. Neurosci. Lett..

[9] Doyle K.P., Simon R.P., Stenzel-Poore M.P. (2008). Mechanisms of ischemic brain damage. Neuropharmacology.

[10] Lo E.H., Moskowitz M.A., Jacobs T.P. (2005). Exciting, radical, suicidal: How brain cells die after stroke. Stroke.

[11] Calabresi P., Saulle E., Centonze D., Pisani A., Marfia G.A., Bernardi G. (2002). Post-ischaemic long-term synaptic potentiation in the striatum: A putative mechanism for cell type-specific vulnerability. Brain.

[12] Calabresi P., Centonze D., Pisani A., Cupini L.M., Bernardi G. (2003). Synaptic plasticity in the ischaemic brain. Lancet Neurol..

[13] Calabresi P., Cupini L.M., Centonze D., Pisani F., Bernardi G. (2003). Antiepileptic drugs as a possible neuroprotective strategy in brain ischemia. Ann. Neurol..

[14] Karten B., Peake K.B., Vance J.E. (2009). Mechanisms and consequences of impaired lipid trafficking in Niemann-Pick type C1-deficient mammalian cells. Biochim. Biophys. Acta.

[15] Rudolphi K.A., Schubert P., Parkinson F.E., Fredholm B.B. (1992). Neuroprotective role of adenosine in cerebral ischaemia. Trends Pharmacol. Sci..

[16] Sweeney M.I. (1997). Neuroprotective effects of adenosine in cerebral ischemia: Window of opportunity. Neurosci. Biobehav. Rev..

[17] Pearson T., Frenguelli B.G. (2000). Volume-regulated anion channels do not contribute extracellular adenosine during the hypoxic depression of excitatory synaptic transmission in area CA1 of rat hippocampus. Eur. J. Neurosci..

[18] Zhou S.Y., Xu S.J., Yan Y.G., Yu H.M., Ling S.C., Luo J.H. (2011). Decreased purinergic inhibition of synaptic activity in a mouse model of Niemann-Pick disease type C. Hippocampus.

[19] Malenka R.C., Nicoll R.A. (1999). Long-term potentiation—A decade of progress?. Science.

[20] D’Arcangelo G., Grossi D., de Chiara G., de Stefano M.C., Cortese G., Citro G., Rufini S., Tancredi V., Merlo D., Frank C. (2011). Glutamatergic neurotransmission in a mouse model of Niemann–Pick Type C Disease. Brain Res..

[21] Frank C., Rufini S., Tancredi V., Forcina R., Grossi D., D’arcangelo G. (2008). Cholesterol depletion inhibits synaptic transmission and synaptic plasticity in rat hippocampus. Exp. Neurol..

[22] Hering H., Lin C.C., Sheng M. (2003). Lipid rafts in the maintenance of synapses, dendritic spines, and surface AMPA receptor stability. J. Neurosci..

[23] Rufini S., Grossi D., Luly P., Tancredi V., Frank C., D’Arcangelo G. (2009). Cholesterol depletion inhibits electrophysiological changes induced by anoxia in CA1 region of rat hippocampal slices. Brain Res..

[24] Liu J. (2014). LOX-1 and neurodegeneration. Neurosci. Lett..

[25] Chen M., Masaki T., Sawamura T. (2002). LOX-1, the receptor for oxidized low-density lipoprotein identified from endotelial cells: Implications in endotelial dysfunction and atherosclerosis. Pharmacol. Ther..

[26] Loftus S.K., Morris J.A., Carstea E.D., Gu J.Z., Cummings C., Brown A., Ellison J., Ohno K., Rosenfeld M.A., Tagle D.A. (1997). Murine model of Niemann–Pick C disease: Mutation in a cholesterol homeostasis gene. Science.

